# A Complex Interplay between Personality Domains, Marital Status and a Variant in *CHRNA5* on the Risks of Cocaine, Nicotine Dependences and Cocaine-Induced Paranoia

**DOI:** 10.1371/journal.pone.0049368

**Published:** 2013-01-07

**Authors:** Tetyana Zayats, Bao-Zhu Yang, Pingxing Xie, James Poling, Lindsay A. Farrer, Joel Gelernter

**Affiliations:** 1 Department of Psychiatry, Yale University School of Medicine, New Haven, Connecticut, United States of America; 2 VACT Healthcare Center, West Haven, Connecticut, United States of America; 3 Departments of Medicine, Neurology, Ophthalmology, Genetics and Genomics, Biostatistics and Epidemiology, Boston University Schools of Medicine and Public Health, Boston, Massachusetts, United States of America; 4 Departments of Neurobiology and Genetics, Yale University School of Medicine, New Haven, Connecticut, United States of America; Tokyo Metropolitan Institute of Medical Science, Japan

## Abstract

**Background:**

Personality correlates highly with both cocaine and nicotine dependencies (CD, ND), and their co-morbid psychopathologies. However, little is known about the nature of these relationships. This study examined if environment (marriage) or genetics (a single SNP, CHRNA5*rs16969968*)* would moderate the correlation of personality with CD, ND and cocaine-induced paranoia (CIP) in African and European Americans (AAs, EAs).

**Methods:**

1432 EAs and 1513 AAs were examined using logistic regression. Personality was assessed by NEO-PI-R, while CD, ND and CIP were diagnosed according to DSM-IV. ND and CD were examined as binary traits and for the analysis of CIP, subjects were divided into 3 groups: (**A**) Controls with no CIP; (**B**) CD cases without CIP; and (**C**) CD cases with CIP. Multiple testing was Bonferroni-corrected.

**Results:**

For CD and ND in the EA population, marital status proved to be a significant moderator in their relationship with *openness* only (OR = 1.90, 95%CI = 1.36–2.64, p = 1.54e-04 and OR = 2.12, 95%CI = 1.52–2.90, p = 4.65e-06 respectively). For CIP, marriage was observed to moderate its correlation with *openness* and *neuroticism* (OR = 1.39, 95%CI = 1.18–1.63, p = 7.64e-04 and OR = 1.26, 95%CI = 1.12–1.42, p = 1.27e-03 respectively). The correlations moderated by rs16969968 were those of *conscientiousness* and CD (OR = 1.62, 95%CI: 1.23–2.12, p = 8.94e-04) as well as CIP (OR = 1.21, 95%CI: 1.11–1.32, p = 4.93e-04 when comparing group A versus group C). No significant interactions were observed in AA population. The Bonferroni-corrected significance threshold was set to be 1.67e-03.

**Conclusion:**

The role of personality in CD and CIP may be interceded by both environment and genetics, while in ND by environment only.

## Introduction

Substance use disorders pose a significant problem for society, with serious health-related, personal and economic consequences [1]–[3]. According to the National Epidemiologic Survey on Alcohol and Related Conditions, approximately 10% of adults in the US experienced a drug-use disorder during their lifetimes [4] and about 20% are smoking cigarettes [5].

One of the most commonly exploited substances is nicotine [6] and it is frequently used in combination with other substances including cocaine [6]–[9]. Both nicotine and cocaine have been shown to act as excitants for each other: the use of cocaine reportedly elevates nicotine intake [10]–[12], while tobacco smoking increases both the frequency and the dosage of cocaine administration [7], [10]. Cocaine and nicotine dependences (ND and CD) are both known to be highly correlated with personality measures. In addition, they also share similarities in their etiological factors, such as marital status and *CHRNA5*.

Personality characteristics have been consistently associated with substance use disorders. Although the hypothesis of “addictive personality” is not well supported [13], [14], it is still unclear whether different personality traits predict distinct forms of drug dependence and whether the effect of personality would still hold if co-morbid psychopathology were accounted for. In general, substance users can be characterized by higher impulsivity, aggression, disinhibition and novelty seeking [15], [16] as well as by high neuroticism, low agreeableness and low conscientiousness [17], [18]. Similarly, nicotine and cocaine dependences have both been found to be associated with neuroticism, agreeableness and conscientiousness [19]–[21]. In addition, cigarette smoking has been linked to extraversion [21]–[23] and CD to openness [17].

Marriage has been cited as a reproducible protective factor against drug use, with rates of abuse and dependence being higher among individuals who are divorced or separated or have never been married [4], [24]. Cessation of cocaine use as well as cigarette smoking has also been linked to one's marital status [21], [25]–[30], showing that being married maintains its preservative effect on these two dependencies too.

Biologically, one of the most replicable genetic risk factors for ND is rs16969968, a non-synonymous SNP in *CHRNA5* [31]–[45]. This polymorphism leads to the substitution of aspartic acid with asparagine (Asp398Asn), which has proven to be functionally relevant in the activity of nicotinic acetylcholine receptor's (nAChR) alpha 5 subunit [41]. In addition, human mRNA clones and expressed sequence tags display evidence of alternative splicing of *CHRNA5* in exon 5, in which rs16969968 resides. Possibly, a decrease in inclusion of rs16969968 in the mature mRNA could decrease the risk for ND [37]. Furthermore, rs16969968 may also act together with the variability in *CHRNA5*'s mRNA expression in human brain: occurrence of non-risk allele of rs16969968 on the background of the low mRNA expression allele of *CHRNA5* leads to a significantly lower risk for cigarette smoking compared to its co-occurrence with the high expression allele [46].

Apart from being functionally relevant and consistently associated with ND, rs16969968 has also been associated with dependence on cannabis [36], alcohol [36], [47], opiates [48], [49] and cocaine [32], [49]. This shared genetic vulnerability of nicotine and cocaine addictions is particularly interesting as the risk for these two dependencies is conferred by the opposite alleles of rs16969968 [32], [49].

Substance dependence traits cannot be explained solely by either personality or by genetic and environmental factors. The purpose of this study was to investigate the complex interplay among personality measures and the elements of environment (marriage) and genetics (*CHRNA5*). The main emphasis was placed on the relationship between personality and CD, ND as well as co-morbid psychopathology in the form of CIP; we hypothesized that marital status and *CHRNA5* could act as moderators in these correlations. Firstly, the possible moderating effect of marriage was examined in the relationship of personality and CD, ND. The alternate explanation for the association between these dependences and personality domains is that it could be altered by the protective environment a marriage may provide: people who get divorced may have similar traits of personality to those who develop CD and ND, making marital status a moderating factor in the correlation between personality and substance dependence. Thus, we postulated that the association of CD and ND with personality measures may not be independent from marital status.

Secondly, the intermediate role of *CHRNA5* SNP rs16969968 was also evaluated in the relationship of the personality measures with CD and ND. Rs16969968 has consistently been associated with both cocaine and nicotine dependences, while personality traits have a strong relationship with the same traits. Since both rs16969968 and personality are associated with ND and CD, there is a possibility of interaction between these two explanatory factors.

Finally, on the account of co-morbid psychopathology predisposing cocaine-dependent patients to developing such specific symptoms as paranoia [50], [51], possible interplay between personality and *CHRNA5* as well as marital status was additionally examined in participants displaying symptoms of cocaine-induced paranoia (CIP).

## Materials and Methods

### Subject recruitment and assessment

Recruitment was conducted at four sites: 1437 participants recruited at the University ofConnecticut Health Center (UCONN, Farmington, CT), 885 participants at Yale University School of Medicine (New Haven, CT), 242 participants at the University of Pennsylvania School of Medicine (UPENN, Philadelphia, PA) and 382 participants at the Medical University of South Carolina (MUSC, Charleston, SC). The protocol and consent forms were approved by the institutional review boards at each site: the UCONN institutional review board, Yale University human research protection program, UPENN office of regulatory affairs- institutional review board and MUSC institutional review board of human research. Written informed consent was obtained from all participants.

All subjects were assessed with the Semi-Structured Assessment for Drug Dependence and Alcoholism (SSADDA) [52], [53], which yields a DSM-IV based lifetime diagnoses for a variety of psychiatric and substance use disorders. Individuals with a primary diagnosis of bipolar affective disorder or schizophrenia were excluded.

Personality traits were characterized in terms of five factors based on the NEO (the Big Five Model-BFM), which comprise behavioral, emotional and cognitive patterns: Neuroticism, Extraversion, Openness to new experiences, Agreeableness and Conscientiousness [54], [55]. The BFM has a hierarchical construction, where each of the five domains includes six facets. The NEO-PI-R (NEO-PI-R) [56] was applied to evaluate these facets of participants' personality.

NEO-PI-R scores were normalized with respect to age and sex of participants as women generally score higher than men on Neuroticism and Agreeableness, while college-age men and women tend to score higher on Openness, Neuroticism, and Extraversion, and lower on Conscientiousness, than do older individuals [57].

Population assignment as either African American (AA) or European American (EA) was done empirically by ancestry proportions estimated in STRUCTURE on the basis of ancestry-informative marker genotypes [58].

### Genotyping

SNP rs16969968 was genotyped with a standard TaqMan technique (assay-on-demand), using the ABI PRISM1 7900 Sequence Detection System (ABI, Foster City, CA). All genotyping was performed in duplicate and compared to ensure validity of the data. Mismatched genotypes were discarded.

### Statistical analyses

All relationships were examined using logistic regression or multinomial logistic regression models adjusted for age and sex. Subjects classified as abusers (as opposed to dependent) on a particular substance were excluded from all analyses for that substance. To avoid confounding due to population stratification, AA and EA subjects were analyzed separately.

ND and CD were coded as dichotomous variables, dividing participants into cases and comparison subjects (controls) and allowing the use of binomial logistic regression for their analyses. The CD affected group consisted of CD patients regardless of their ND status; likewise, the ND. Since CD and ND were the foci of the analyses, these two dependencies only were considered in the definition of “control” and only those individuals with neither of these diagnoses were classified into this group.

For the multinomial logistic regression analysis of CIP, CD cases (including those co-morbid with ND) and controls were divided into 3 groups: (A) controls; (B) CDs with no CIP symptoms; (C) CDs exhibiting CIP symptoms. Cases with nicotine dependence only were excluded from these analyses. Marital status was coded as (1) never married, (2) separated or divorced and (3) married. Subjects who were widowed at the time of recruitment were excluded (because this would not reasonably be expected to correlate with the subject's personality). Rs16969968 genotype was coded as a dichotomous variable depending on whether an individual carried at least one copy of the minor “A” allele of this SNP.

Before examining the possibility of a moderating effect of marital status and rs16969968 on the role of personality in CD, ND and CIP, exploratory logistic regression was performed to evaluate if there was a significant correlation between personality domains and CD, ND and CIP in our sample at all. In addition, Spearman correlations between all explanatory variables (personality measures, marital status and rs16969968 genotype) were also examined in order to avoid potential co-linearity.

All of the five personality measures revealed significant correlation with CD, ND and CIP; and because these dimensions are known not to be independent from each other, each personality domain was evaluated separately, culminating in five regressions examined for each of the outcomes of CD, ND and CIP. Since none of the explanatory variables showed high correlation with each other (all Spearman's rho values <0.25), regression models were constructed to include the main effects of personality domain (one at a time), marital status and rs16969968, and all of the pair-wise two-way interactions. After evaluating the two-way interactions, the interplay between personality, marital status and rs16969968 in CD, ND and CIP was examined with saturated models by adding a three-way interaction term (personality domain x marital status x rs16969968).

Adjustment for multiple testing was done by Bonferroni correction. Since personality scores were not independent, Bonferroni correction was performed based on the number of autonomous tests only: three models for outcomes of CD, ND, and CIP with two-way interactions, and another three saturated models including the three-way interaction. In addition, the overall significance threshold was reduced to a more stringent level of 0.01, to avoid false positive results. Thus, the Bonferroni-corrected significance threshold p-value was 1.67e-03 (0.01/6) in this study.

## Results

### Subject recruitment and assessment

In total, 2946 (1432 EA and 1514 AA) unrelated subjects were included. Among EAs, the average age was 38.3 (SD = 11.7) years and 46.9% of individuals were females. Among AAs, the average age was 41.3 (SD = 9.08) years and 44.4% of participants were females. Information on lifetime substance dependence diagnoses is summarized in Table 1.

**Table 1:** Summary of DSM-IV based, life-time diagnoses of substance addictions in study participants.

	Number of Individuals	
	European Americans	African Americans
**Cocaine Dependence**		
Cases	1014	1178
controls	418	335
Co-morbid ND in cases	786 (77.51%)	762 (64.68%)
Co-morbid ND in controls	None	None
Co-morbid AD in cases	639 (63.02%)	657 (55.77%)
Co-morbid AD in controls	59 (14.11%)	55 (16.42%)
Co-morbid OD in cases	697 (68.74%)	312 (26.48%)
Co-morbid OD in controls	34 (8.13%)	19 (5.67%)
Co-morbid MDD in cases	208 (20.51%)	162 (13.75%)
Co-morbid MDD in controls	36 (8.61%)	29 (8.66%)
**Nicotine Dependence**		
Cases	1029	845
controls	418	335
Co-morbid CD in cases	786 (76.38%)	762 (90.28%)
Co-morbid CD in controls	None	None
Co-morbid AD in cases	630 (61.22%)	510 (60.35%)
Co-morbid AD in controls	59 (14.11%)	247 (73.73%)
Co-morbid OD in cases	718 (69.78%)	260 (30.77%)
Co-morbid OD in controls	34 (8.13%)	103 (30.75%)
Co-morbid MDD in cases	213 (20.70%)	130 (15.38%)
Co-morbid MDD in controls	36 (8.61%)	72 (21.49%)
**Cocaine-Induced Paranoia**		
CD cases without CIP	328	364
CD cases with CIP	686	814
controls	418	335
Co-morbid ND in cases without CIP	242 (73.78%)	226 (62.09%)
Co-morbid ND in cases with CIP	544 (79.30%)	536 (65.85%)
Co-morbid ND in controls	None	None
Co-morbid AD in cases without CIP	186 (56.71%)	173 (47.53%)
Co-morbid AD in cases with CIP	453 (66.03%)	484 (59.46%)
Co-morbid AD in controls	59 (14.11%)	55 (16.42%)
Co-morbid OD in cases without CIP	228 (69.51%)	98 (26.92%)
Co-morbid OD in cases with CIP	469 (68.37%)	214 (26.29%)
Co-morbid OD in controls	34 (8.13%)	19 (5.67%)
Co-morbid MDD in cases without CIP	61 (18.60%)	49 (13.46%)
Co-morbid MDD in cases with CIP	147 (21.43%)	113 (13.88%)
Co-morbid MDD in controls	36 (8.61%)	29 (8.66%)

CD = cocaine dependence, ND = nicotine dependence, AD = alcohol dependence, OD = opiate dependence, MDD = major depression disorder, CIP = cocaine induced paranoia.

The scores of main five domains in NEO-PI-R followed normal distribution. Their mean and standard deviations are presented in Table 2.

**Table 2:** Mean and standard deviation (SD) of NEO-PI-R normalized scores in the participants of the study.

European American Population					
	Mean (Standard Deviation) score of				
	Neuroticism	Extraversion	Openness to experience	Agreeableness	Conscientiousness
CD Cases	60.15 (9.63)	48.60 (9.61)	47.63 (9.12)	41.20 (10.38)	38.77 (10.81)
CD Controls	51.51 (11. 12)	50.92 (10.45)	50.69 (10.07)	47.89 (12.29)	46.96 (12.30)
ND Cases	59.99 (9.51)	48.22 (9.49)	47.71 (9.21)	51.29 (10.63)	38.69 (10.75)
ND Controls	48.61 (10.59)	52.21 (10.66)	51.39 (10.15)	50.49 (11.79)	50.28 (11.72)
CD cases without CIP	58.18 (9.84)	48.64 (9.78)	47.36 (8.99)	42.13 (9.98)	39.29 (11.08)
CD cases with CIP	61.08 (9.39)	48.58 (9.54)	47.75 (9.19)	40.76 (10.55)	38.53 (10.68)
CIP Controls (no CD and no CIP)	48.61 (10.59)	52.21 (10.66)	51.39 (10.14)	50.49 (11.79)	50.28 (11.72)

African American Population					
	Mean (Standard Deviation) score of				
	Neuroticism	Extraversion	Openness to experience	Agreeableness	Conscientiousness
CD Cases	55.51 (7.93)	47.94 (7.92)	46.33 (7.71)	41.08 (9.63)	42.61 (9.70)
CD Controls	47.94 (8.44)	50.63 (9.01)	48.69 (8.49)	48.84 (10.54)	51.44 (9.99)
ND Cases	55.91 (7.97)	47.70 (7.82)	46.19 (7.66)	40.84 (9.73)	42.14 (9.59)
ND Controls	51.52 (8.75)	49.34 (8.59)	47.54 (8.13)	44.89 (10.58)	47.36 (10.63)
CD cases without CIP	54.89 (8.25)	47.68 (8.00)	46.25 (7.85)	41.58 (9.55)	43.08 (10.14)
CD cases with CIP	55.79 (7.77)	48.05 (7.89)	46.36 (7.65)	40.85 (9.66)	42.39 (9.49)
CIP Controls (no CD and no CIP)	47.94 (8.44)	50.63 (9.01)	48.69 (8.49)	48.84 (10.54)	51.44 (9.99)

CD = cocaine dependence, ND = nicotine dependence, CIP = cocaine induced paranoia.

### Genotyping of rs16969968

All included subjects were successfully genotyped for rs16969968. Consistent with previous reports (minor allele frequency ranges from 33% to 43% in EA; and ∼5% in AA [31], [32], [41]), the observed minor allele frequency for this SNP was 35.3% in EA subjects and 6.6% in AA participants. Genotype distributions were consistent with Hardy-Weinberg equilibrium expectations in both populations overall, as well as in case and control groups of AAs and EAs.

### Statistical analyses

For examining the interaction between personality measures and marital status, significant moderating effects of both marriage and divorce were observed in the relationship between the “*openness to experience”* domain and CD, ND as well as CIP in the EA population only (Tables 3, 4 and 5). The strongest impact appeared to be generated by “married” status compared to the “never married” state on the correlations between “*openness to experience*” and ND (OR: 2.12, 95%CI: 1.36–2.64, p-value: 4.65e-06), followed by CD (OR: 1.90, 95%CI:1.55–2.90, p-value:1.54e-04) and CIP (OR: 1.39, 95%CI: 1.18–1.63, p-value: 7.64e-04).

**Table 3:** Effect of marital status and *CHRNA5* (rs16969968) on the relationship between NEO-PI-R personality domains and CD in AA and EA subjects.

		NEO-PI-R domain and Population									
		Neuroticism		Extraversion		Openness to experience		Agreeableness		Conscientiousness	
		AAs	EAs	AAs	EAs	AAs	EAs	AAs	EAs	AAs	EAs
Main effect of NEO-PI-R domain	OR (95% CI)	**3.00 (2.33–3.88)**	**2.91 (2.22–3.83)**	**0.66 (0.53–0.82)**	**0.54 (0.42–0.68)**	**0.59 (0.48–0.74)**	**0.44 (0.34–0.57)**	**0.51 (0.50–0.52)**	**0.43 (0.34–0.55)**	**0.43 (0.35–0.52)**	**0.30 (0.23–0.39)**
	p-value	**2.00e-16**	**1.40e-14**	**1.70e-04**	**4.60e-07**	**3.28e-06**	**1.65e-10**	**5.90e-12**	**4.69e-12**	**2.00e-16**	**2.00e-16**
Main effect of “Divorced” marital status	OR (95% CI)	1.03 (0.81–1.32)	1.09 (0.89–1.33)	0.96 (0.77–1.21)	0.81 (0.68–0.95)	0.75 (0.60–0.94)	0.76 (0.64–0.91)	1.17 (0.97–1.42)	1.08 (0.91–1.27)	0.94 (0.78–1.13)	0.94 (0.80–1.11)
	p-value	0.780	0.389	0.767	0.011	0.013	2.27e–03	0.100	0.368	0.496	0.475
Main effect of “Married” marital status	OR (95% CI)	0.84 (0.65–1.08)	**0.64 (0.51–0.82)**	0.86 (0.70–1.05)	**0.72 (0.61–0.85)**	0.77 (0.63–0.95)	**0.62 (0.53–0.74)**	1.06 (0.84–1.20)	0.98 (0.83–1.16)	1.03 (0.84–1.28)	0.97 (0.825–1.14)
	p-value	0.180	**2.72e-04**	0.147	**1.21e-04**	0.013	**4.43e-08**	0.947	0.859	0.750	0.736
Main effect of rs16969968	OR (95% CI)	0.77 (0.54–1.09)	1.01 (0.85–1.34)	0.81 (0.64–1.03)	0.95 (0.83–1.10)	0.94 (0.73–1.21)	0.93 (0.80–1.07)	1.01 (0.99–1.03)	0.92 (0.82–1.05)	1.03 (0.81–1.31)	**0.80 (0.70–0.91)**
	p-value	0.136	0.886	0.083	0.507	0.656	0.317	0.953	0.225	0.795	**1.05-03**
Interaction between NEO-PI-R domain and “Divorced” marital status	OR (95% CI)	0.92 (0.57–1.51)	0.88 (0.62–1.25)	1.05 (0.67–1.64)	1.65 (1.20–2.26)	1.77 (1.13–2.77)	**1.80 (1.29–2.52)**	0.71 (0.49–1.03)	0.95 (0.68–1.31)	1.14 (0.79–1.65)	1.16 (0.83–1.62)
	p-value	0.756	0.466	0.845	2.02e-03	0.015	**5.24e-04**	0.085	0.734	0.499	0.351
Interaction between NEO-PI-R domain and “Married” marital status	OR (95% CI)	1.18 (0.69–1.97)	1.91 (1.24–2.95)	1.09 (0.72–1.65)	1.45 (1.06–1.98)	1.35 (0.88–2.08)	**1.90 (1.36–2.64)**	0.79 (0.55–1.15)	0.80 (0.57–1.12)	0.75 (0.49–1.15)	0.81 (0.58–1.13)
	p-value	0.505	3.58e-03	0.676	0.020	0.169	**1.54e-04**	0.239	0.206	0.185	0.218
Interaction between NEO-PI-R domain and rs16969968	OR (95% CI)	1.73 (0.87–3.44)	0.96 (0.72–1.29)	1.58 (0.99–2.53)	1.08 (0.84–1.40)	1.17 (0.70–1.95)	1.90 (1.44–2.49)	1.03 (0.67–1.58)	1.18 (0.92–1.53)	0.95 (0.60–1.53)	**1.62 (1.23–2.12)**
	p-value	0.111	0.817	0.056	0.534	0.538	0.383	0.887	0.206	0.850	**8.94e-04**
Interaction between rs16969968 and “Divorced” marital status	OR (95% CI)	1.05 (0.91–1.21)	1.03 (0.96–1.10)	1.00 (0.90–1.12)	1.02 (0.96–1.09)	1.01 (0.90–1.13)	1.03 (0.97–1.10)	1.00 (0.89–1.12)	1.01 (0.94–1.08)	0.97 (0.87–1.09)	1.04 (0.97–1.11)
	p-value	0.464	0.419	0.975	0.500	0.841	0.326	0.981	0.830	0.668	0.299
Interaction between rs16969968 and “Married” marital status	OR (95% CI)	0.98 (0.92–1.04)	1.01 (0.94–1.08)	1.00 (0.89–1.11)	1.03 (0.97–1.09)	1.00 (0.90–1.11)	1.04 (0.98–1.11)	0.96 (0.86–1.08)	1.01 (0.95–1.08)	0.95 (0.85–1.07)	1.03 (0.96–1.10)
	p-value	0.755	0.853	0.951	0.389	0.968	0.193	0.551	0.681	0.407	0.456

Reference group for marital status were subjects who have never been married. Reference group for the genotypes of rs16969968 were subjects who did not have any copies of minor allele. All odds ratios (OR) refer to 10 units change in personality scores, meaning that the presented odds of an individual developing CD reflect the NEO-PI-R domain score change in the magnitude of 10. Presented p-values are not adjusted for multiple testing. Bonferroni-corrected significance threshold was set to 1.67e-03. Significant p-values are highlighted in bold.

**Table 4:** Effect of marital status and *CHRNA5* (rs16969968) on the relationship between NEO-PI-R personality domains and ND in AA and EA subjects.

		NEO-PI-R domain and Population									
		Neuroticism		Extraversion		Openness to experience		Agreeableness		Conscientiousness	
		AAs	EAs	AAs	EAs	AAs	EAs	AAs	EAs	AAs	EAs
Main effect of NEO-PI-R domain	OR (95% CI)	**3.22 (2.45–4.24)**	**2.89 (2.19–3.80)**	**0.63 (0.51–0.78)**	**0.58 (0.46–0.74)**	**0.57 (0.45–0.72)**	**0.45 (0.35–0.59)**	**0.50 (0.41–0.60)**	**0.47 (0.37–0.59)**	**0.41 (0.33–0.51)**	**0.32 (0.25–0.41)**
	p-value	**2.00e-16**	**2.26e-14**	**6.13e-05**	**7.57e-06**	**3.10e-06**	**3.87e-10**	**1.39e-11**	**7.47e-11**	**5.16e-16**	**2.00e-16**
Main effect of “Divorced” marital status	OR (95% CI)	1.02 (0.78–1.32)	1.09 (0.89–1.34)	1.00 (0.79–1.27)	0.85 (0.72–1.00)	0.75 (0.59–0.94)	0.76 (0.64–0.91)	1.20 (0.98–1.47)	1.09 (0.92–1.28)	0.92 (0.76–1.11)	0.96 (0.81–1.12)
	p-value	0.895	0.381	0.898	0.057	0.014	2.82e-03	0.074	0.305	0.400	0.593
Main effect of “Married” marital status	OR (95% CI)	0.85 (0.65–1.12)	**0.64 (0.50–0.81)**	0.86 (0.68–1.08)	0.77 (0.65–0.91)	0.76 (0.61–0.94)	**0.59 (0.50–0.70)**	1.02 (0.84–1.23)	0.96 (0.81–1.13)	1.12 (0.88–1.43)	1.01 (0.85–1.20)
	p-value	0.258	**2.77e-04**	0.188	2.29e-03	0.012	**6.52e-10**	0.873	0.612	0.357	0.886
Main effect of rs16969968	OR (95% CI)	0.82 (0.58–1.15)	0.98 (0.82–1.16)	0.77 (0.60–0.99)	1.01 (0.88–1.16)	0.91 (0.70–1.17)	0.93 (0.81–1.08)	0.97 (0.79–1.19)	0.96 (0.85–1.09)	1.04 (0.80–1.34)	0.84 (0.73–0.96)
	p-value	0.250	0.777	0.041	0.875	0.450	0.357	0.771	0.542	0.763	9.10e-03
Interaction between NEO-PI-R domain and “Divorced” marital status	OR (95% CI)	0.96 (0.58–1.60)	0.88 (0.62–1.25)	1.00 (0.62–1.60)	1.46 (1.07–2.00)	1.79 (1.12–2.86)	**1.77 (1.27–2.47)**	0.67 (0.44–1.01)	0.91 (0.66–1.24)	1.17 (0.81–1.70)	1.12 (0.80–1.56)
	p-value	0.876	0.462	0.997	0.020	0.017	**8.48e-04**	0.057	0.559	0.394	0.517
Interaction between NEO-PI-R domain and “Married” marital status	OR (95% CI)	1.14 (0.67–1.93)	1.97 (1.26–3.10)	1.08 (0.69–1.70)	1.27 (0.93–1.74)	1.40 (0.90–2.21)	**2.12 (1.55–2.90)**	0.77 (0.51–1.16)	0.85 (0.62–1.17)	0.63 (0.38–1.02)	0.76 (0.54–1.09)
	p-value	0.640	3.23e-03	0.737	0.129	0.143	**4.65e-06**	0.212	0.325	0.068	0.131
Interaction between NEO-PI-R domain and rs16969968	OR (95% CI)	1.60 (0.81–3.18)	1.05 (0.77–1.44)	1.80 (1.15–2.83)	0.97 (0.75–1.25)	1.31 (0.79–2.18)	1.12 (0.85–1.47)	1.16 (0.75–1.79)	1.09 (0.85–1.41)	0.97 (0.58–1.62)	1.48 (1.12–1.94)
	p-value	0.173	0.757	0.021	0.828	0.305	0.391	0.502	0.491	0.918	6.15e-03
Interaction between rs16969968 and “Divorced” marital status	OR (95% CI)	1.03 (0.91–1.17)	1.02 (0.95–1.09)	1.02 (0.91–1.13)	1.02 (0.96–1.09)	1.03 (0.91–1.15)	1.03 (0.97–1.10)	1.00 (0.89–1.13)	1.01 (0.94–1.08)	0.98 (0.87–1.10)	1.03 (0.97–1.11)
	p-value	0.626	0.574	0.771	0.543	0.661	0.368	0.970	0.789	0.717	0.347
Interaction between rs16969968 and “Married” marital status	OR (95% CI)	1.02 (0.90–1.15)	1.01 (0.94–1.08)	1.01 (0.90–1.13)	1.01 (0.95–1.08)	1.01 (0.90–1.12)	1.03 (0.97–1.09)	0.99 (0.88–1.11)	1.01 (0.94–1.07)	0.96 (0.85–1.08)	1.00 (0.93–1.07)
	p-value	0.811	0.846	0.879	0.662	0.903	0.387	0.845	0.839	0.478	0.998

Reference group for marital status were subjects who have never been married. Reference group for the genotypes of rs16969968 were subjects who did not have any copies of minor allele. All odds ratios (OR) refer to 10 units change in personality scores, meaning that the presented odds of an individual developing ND reflect the NEO-PI-R domain score change in the magnitude of 10. Presented p-values are not adjusted for multiple testing. Bonferroni-corrected significance threshold was set to 1.67e-03. Significant p-values are highlighted in bold.

**Table 5:** Effect of marital status and *CHRNA5* (rs16969968) on the relationship between NEO-PI-R personality domains and CIP in AA and EA subjects.

		NEO-PI-R domain and Population									
		Neuroticism		Extraversion		Openness to experience		Agreeableness		Conscientiousness	
		AAs	EAs	AAs	EAs	AAs	EAs	AAs	EAs	AAs	EAs
Main effect of NEO-PI-R domain	OR (95% CI)	**1.57 (1.49–1.65)**	**1.60 (1.47–1.74)**	**0.84 (0.80–0.89)**	**0.76 (0.69–0.84)**	**0.79 (0.76–0.83)**	**0.70 (0.63–0.78)**	**0.78 (0.74–0.82)**	**0.70 (0.65–0.77)**	**0.72 (0.69–0.75)**	**0.64 (0.59–0.69)**
	p-value	**2.00e-16**	**2.00e-16**	**5.28e-04**	**1.65e-05**	**1.57e-05**	**4.75e-08**	**9.49e-10**	**4.19e-11**	**2.00e-16**	**2.00e-16**
Main effect of “Divorced” marital status	OR (95% CI)	1.10 (1.05–1.16)	1.03 (0.96–1.10)	0.92 (0.88–0.97)	0.92 (0.86–0.98)	0.88 (0.84–0.92)	0.87 (0.81–0.94)	0.99 (0.95–1.04)	0.99 (0.94–1.05)	0.92 (0.87–0.96)	0.95 (0.90–0.99)
	p-value	0.031	0.502	0.101	0.035	8.99e-03	1.55e-03	0.848	0.821	5.97e-03	0.085
Main effect of “Married” marital status	OR (95% CI)	0.93 (0.88–0.97)	**0.85 (0.79–0.90)**	0.92 (0.88–0.97)	0.90 (0.83–0.97)	0.89 (0.85–0.93)	**0.78 (0.72–0.85)**	1.08 (1.03–1.13)	1.00 (0.94–1.07)	1.04 (0.99–1.09)	1.02 (0.96–1.07)
	p-value	0.157	**3.90e-05**	0.154	0.30	0.028	**6.97e-07**	0.051	0.953	0.371	0.634
Main effect of rs16969968	OR (95% CI)	0.98 (0.93–1.03)	1.00 (0.95–1.06)	0.93 (0.88–0.97)	0.95 (0.89–1.00)	0.86 (0.91–1.01)	0.96 (0.90–1.02)	1.00 (0.95–1.05)	0.96 (0.92–1.01)	1.03 (0.98–1.08)	**0.91 (0.88–0.95)**
	p-value	0.705	0.930	0.173	0.112	0.028	0.223	0.985	0.165	0.475	**1.38e-04**
Interaction between NEO-PI-R domain and “Divorced” marital status	OR (95% CI)	0.84 (0.80–0.88)	0.95 (0.85–1.07)	1.17 (1.12–1.23)	1.22 (1.07–1.39)	1.30 (1.24–1.36)	**1.35 (1.18–1.55)**	1.01 (0.96–1.06)	1.05 (0.94–1.17)	1.22 (1.16–1.28)	1.13 (1.02–1.26)
	p-value	0.027	0.477	0.108	0.0127	8.98e-03	**4.56e-04**	0.871	0.470	4.69e-03	0.057
Interaction between NEO-PI-R domain and “Married” marital status	OR (95% CI)	1.10 (1.05–1.16)	**1.26 (1.12–1.42)**	1.08 (1.03–1.13)	1.06 (0.91–1.23)	1.18 (1.12–1.24)	**1.39 (1.18–1.63)**	0.79 (0.75–0.83)	0.88 (0.77–1.00)	0.86 (0.82–0.90)	0.86 (0.77–0.96)
	p-value	0.347	**1.27e-03**	0.485	0.546	0.156	**7.64e-04**	3.91e-03	0.091	0.079	0.024
Interaction between NEO-PI-R domain and rs16969968	OR (95% CI)	1.07 (1.02–1.12)	0.97 (0.89–1.07)	1.21 (1.15–1.27)	1.09 (0.98–1.22)	1.14 (1.08–1.19)	1.06 (0.94–1.19)	1.05 (1.00–1.10)	1.06 (0.97–1.17)	0.96 (092–1.01)	**1.21 (1.11–1.32)**
	p-value	0.521	0.634	0.082	0.209	0.156	0.424	0.605	0.299	0.705	**4.93e-04**
Interaction between rs16969968 and “Divorced” marital status	OR (95% CI)	1.00 (0.95–1.05)	1.02 (0.99–1.04)	1.00 (0.95–1.05)	1.02 (0.99–1.04)	0.99 (0.94–1.04)	1.02 (0.99–1.04)	0.99 (0.94–1.04)	1.01 (0.98–1.03)	0.99 (0.94–1.04)	1.02 (0.99–1.05)
	p-value	0.999	0.249	0.902	0.353	0.996	0.296	0.697	0.630	0.690	0.191
Interaction between rs16969968 and “Married” marital status	OR (95% CI)	1.00 (0.95–1.05)	1.00 (0.97–1.03)	0.98 (0.93–1.03)	1.02 (0.99–1.05)	0.98 (0.93–1.03)	1.02 (0.99–1.05)	0.99 (0.94–1.04)	1.01 (0.98–1.04)	0.98 (0.94–1.03)	1.00 (0.98–1.03)
	p-value	0.950	0.906	0.945	0.376	0.994	0.215	0.751	0.716	0.541	0.834

Reference group for marital status were subjects who have never been married. Reference group for the genotypes of rs16969968 were subjects who did not have any copies of minor allele. All odds ratios (OR) refer to 10 units change in personality scores, meaning that the presented odds of an individual developing CIP reflect the NEO-PI-R domain score change in the magnitude of 10. Results shown in the table are those of comparison between groups A (controls with no CIP) and C (CDs with CIP). Presented p-values are not adjusted for multiple testing. Bonferroni-corrected significance threshold was set to 1.67e-03. Significant p-values are highlighted in bold.

**Figure 1:**
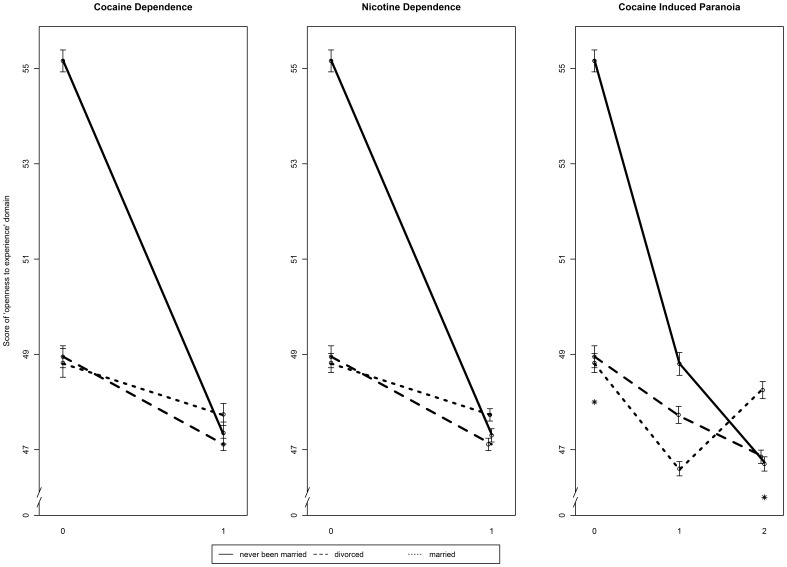
Interaction plot reflecting significant moderation of marital status on correlation between “*openness to experience*” NEO-PI-R domain and the risk of CD, ND and CIP in EAs. The Y axis represents the score of “*openness to experience*” domain, while the X axis shows the phenotype examined (for cocaine and nicotine dependences zero stands for controls and one stands for cases; for cocaine induced paranoia zero stands for controls, one stands for cocaine dependent cases without CIP and two stands for cocaine dependent cases with CIP). The error bars represent standard error of calculated “*openness to experience*” scores in each group of marital status in cases and controls.

Never-marrieds consistently scored higher in the “*openness to experience*” domain compared to either divorced or married participants in control groups for CD, ND and CIP (Figure 1). This pattern was not present among affected individuals; in the substance dependent subjects, divorced and never married subjects appeared to present somewhat lower scores than those who were married, especially in subjects suffering from both CD and CIP (Figure 1).

**Figure 2:**
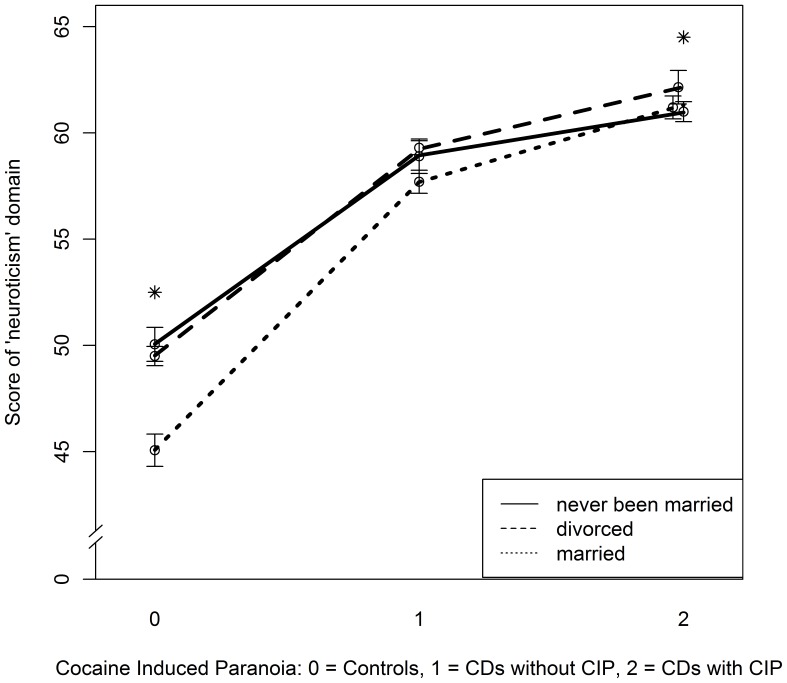
Interaction plot reflecting significant moderation of marital status on correlation between “*neuroticism*” NEO-PI-R domain and the risk of CIP in EAs. The Y axis represents the score of “*neuroticism*” domain, while the X axis shows the categories of CIP: zero stands for controls, one stands for cocaine dependent cases without CIP and two stands for cocaine dependent cases with CIP. The error bars represent standard error of calculated “*neuroticism*” scores in each group of marital status in cases and controls. The asterisks indicate the two groups (controls and CD cases with symptoms of paranoia) that revealed significant interaction (there was no significant result observed between controls and CD cases without symptoms of paranoia or CD cases with and without symptoms of paranoia).

In addition to the observed moderating effect of marital status on the association between “*openness to experience*” and all three examined outcomes (i.e. CD, ND and CIP), a significant interaction also occurred between marriage and the domain of “*neuroticism*” in the development of CIP in the EA population only (OR:1.26, 95%CI:1.12–1.42, p-value:1.27e-03, Table 5). Similarly to the moderating trend marriage displayed on the “*openness to experience”* domain, never-married controls (with neither CD nor CIP) revealed the highest score in “*neuroticism*” domain as well (Figure 2). Cases (with CD and CIP) demonstrated a contrasting pattern, with divorced individuals scoring the highest (Figure 2).

**Figure 3:**
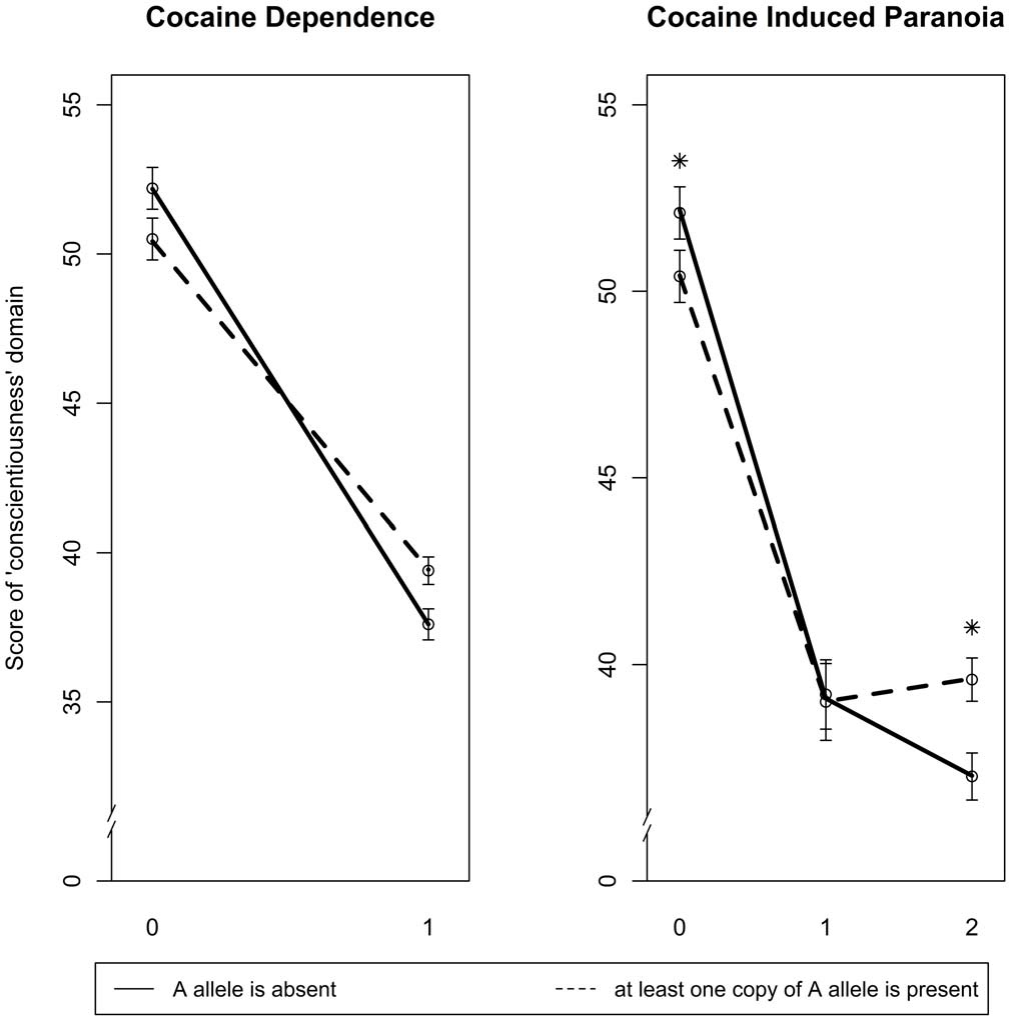
Interaction plot reflecting significant moderation of rs16969968 on correlation between “*conscientiousness”* NEO-PI-R domain and the risk of CD and CIP in EA population. The Y axis represents the score of “conscientiousness” domain, while the X axis shows the phenotype examined (for cocaine and nicotine dependences zero stands for controls and one stands for cases; for cocaine induced paranoia zero stands for controls, one stands for cocaine dependent cases without CIP and two stands for cocaine dependent cases with CIP). The error bars represent standard error of calculated “*conscientiousness*” scores in each group of marital status in cases and controls. The asterisks indicate the two groups (controls and CD cases with symptoms of paranoia) that revealed significant interaction (there was no significant result observed between controls and CD cases without symptoms of paranoia or CD cases with and without symptoms of paranoia).

For evaluating the possibility of interaction between rs16969968 and NEO-PI-R domains, a significant result was noted for the risk of CD and CIP in the EA population only (Table 5). Among subjects with the diagnosis of CD, those with at least one copy of protective allele “A” of rs16969968 were more conscientious (revealed higher score in “*conscientiousness*” domain) than those without it, while reverse scoring was observed in subjects who did not suffer from CD (Figure 3). Similar interaction pattern was revealed in the risk of CIP: individuals who displayed symptoms of CIP in addition to CD scored higher on conscientiousness if they carried at least one rs16969968 “A” allele compared to those without it, with the contrary scores in the control group (Figure 3).

No significant interactions were observed in the AA population.

## Discussion

This study focused on the role of personality and *CHRNA5* variation in CD and ND in both EA and AA populations. The purpose of the analyses described here was to examine potential interaction between NEO-PI-R personality domains with marital status and rs16969968 in *CHRNA5* on the risks of CD, ND and CIP.

Consistent with our hypothesis, the measured personality domains did show interaction with both marital status and rs16969968 in their correlations with CD, ND and CIP, although the observed moderating effect did not extend to every domain and each of the examined moderators interceded in different correlations. In addition, there was no significant interaction observed in AA population.

Firstly, the effect of marital status (considered an environmental factor) was evaluated on the relationship of NEO-PI-R personality domains with CD, ND and CIP. Significant interaction was noted between the “*openness to experience”* domain and marital status on the risks of CD, ND and CIP in the EA population only (Tables 3, 4 and 5). Moreover, a significant moderating effect of marriage was also present in the correlation between “*neuroticism”* and CIP in EAs (Table 5). These observations suggest that the link between personality and CD, ND and CIP is not trivial and may be affected by the surrounding environment.

Secondly, the role of rs16969968 in the association between NEO-PI-R domains with CD, ND and CIP was assessed. This time, it was the domain of “*conscientiousness*” that showed significant interaction with rs16969968 in its correlation with CD and CIP in EA subjects only (Tables 3 and 5). No significant result was observed in relation to ND (Table 4), postulating that such genetic factor as rs16969968 in *CHRNA5* may be acting differently in etiology of ND compared to CD or CIP.

Since the “big five” are dimensions of perceived personality [59] and represent isomorphic depiction of observed behavior, to interpret its meaning fully, the entire chain of causation from the neuropsychiatric bases of behavior in targets to the inferential processes by which perceivers perceive should be examined [60]. The five factors of the NEO-PI-R could be, however, reformulated to accommodate their social-functional significance for perceivers: Denissen and Penke, for example, portray the five-factor model as individual differences in motivational reactions to situations [61]. Similarly, Tellegen as well as Revelle postulate that the five dimensions could be viewed as stable individual differences in people's reactions to circumscribed environmental cues [62]. Thus, although the big five dimensions are conceptually and empirically related, there is a difference between the content of person's motivations and how he or she will try to achieve them [61]. Below we offer a speculation on how our results could be interpreted in terms of those differences.

*“Openness to experience”* involves the tendency to be creative, imaginative, attuned to inner feelings and inclined towards new activities [56]. It could be interpreted, in part, as differences in the activation of the inner reward system when engaging in cognitive activity [61]. Considering marriage or divorce as a situational cue (e.g. presence or absence of stress), the score in the “*openness to experience*” domain may reflect individual differences in how people would react to it. Consistent with this assumption, “*openness to experience*” has been reported to be a correlate of close relationship [63] and to be associated with several marital outcomes [64] as well as marital satisfaction [65]. Moreover, it may be recognized that an individual's coping skills may be an inevitable part of how he or she would react to an environmental cue. Consequently, individuals scoring high in “*openness to experience”* tend to engage in more adaptive, flexible coping [66], while those who score low show greater vulnerability to adverse effects of stress [67]. In this study, controls tended to score higher on the “*openness to experience*” domain, compared to cases (independent of marital status; Figure 1). In addition, married cases appeared to show somewhat higher scores in this domain than never-marrieds, so we hypothesize that not being in a marriage (which could be considered a safe and stable environment) combined with lower scores of “*openness to experience*” and possibly worse coping structures may increase the risk of developing either CD, ND or CIP.

*“Neuroticism”* reflects the disposition to be impulsive and to experience negative emotions such as depression, anxiety or anger [56]. It can be characterized, in part, as differences in activation of the punishment system when faced with the cues of social exclusion [61]. In the sense that specific trajectories of social participation may play an important role in the development of paranoia [68] and marriage may be viewed as a criterion for a certain social inclusions or exclusions, it is easy to see that the “*neuroticism*” domain could interact with marital status in the development of CIP in cocaine dependent subjects. Consistently, subjects who developed symptoms of CIP in addition to CD showed the highest scores of “*neuroticism”* in the divorced group compared to either married or never married individuals (Figure 2), who could be viewed as socially more acceptable than divorced participants.

Since the interaction between NEO-PI-R domains and marital status on the risk of CIP expanded to more personality domains than that on the risk of CD, marriage might have a different moderating effect on personality and co-morbid psychopathology of CD than it has on CD itself.

*“Conscientiousness”* appears to reflect self-control, determination and organization. It encompasses several features that may manifest the measure of socialization in a broad manner, involving individual differences in the behavioral predisposition to follow socially prescribed norms for impulse control, to be goal-directed and be able to follow rules [69]. Thus, individuals scoring high on “*conscientiousness”* tend to use a more active, problem-solving strategies and to avoid emotion-based solutions (e.g. self-blame) [70]. In this study, subjects possessing at least one protective allele “A” of rs16969968 (protective against CD) scored higher on “*conscientiousness”* than those who had no copies of that allele in the group of CD cases and CD cases with CIP (Figure 3).

*CHRNA5* rs16969968 has been previously observed to be involved in phenotype and in gene x environment interaction: it has been reported to interact with age at onset of smoking [71], [72], peer smoking [73] and parent monitoring [74] in the development of ND, as well as relapse likelihood and withdrawal severity [72], cognitive function [75] and BMI [76] in nicotine dependence. These findings refer to nicotine; this study did not observe significant interaction between rs16969968 and NEO-PI-R personality domains on the risk of ND, but on the risk of CD only. Nonetheless, these previously reported moderations along with the one described here, are consistent with the formulation that in the genetics of complex disorders, including substance dependence, individual polymorphisms can generally only account for a small part of phenotypic variance [77]–[79]. Thus, the moderating effect of rs16969968 on the correlation of personality with CD and CIP is small in magnitude.

Since “*conscientiousness”* has been linked to several different drug dependencies (not only cocaine) and it is still uncertain whether the impact of distinct personality traits would be similar on different substance disorders or co-morbid psychopathology, the interaction between rs16969968 and conscientiousness on the risk of CD would need to be evaluated in the light of possible confounding effects of co-occurring addictions. However, this was not plausible within the logistic regression design of this study because of the observed high correlation between CD and co-morbid alcohol dependence (Spearman's rho = 0.599) as well as opioid dependence (Spearman's rho = 0.573).

None of the moderating effects observed in EAs were present in AA individuals. A possible explanation could be that African Americans may demonstrate unique patterns and pathways of substance use and mental health problems compared to other racial/ethnic groups [80], [81]. Individuals of different ethnic descent have been reported to show disparate nicotine intake and metabolism that may account for variability in nicotine use outcomes: European Americans tend to smoke greater numbers of cigarettes when compared with African Americans [82], [83] and African Americans seem to have greater nicotine intake from tobacco smoke [84]. Similarly, inter-ethnic discrepancies in cocaine use related conditions have also been demonstrated. Although it remains unclear whether substance use and psychological distress influence one another over time, cocaine related suicide in teenagers, for example, appear to be less common in African Americans compared to European Americans [85], suggesting inter-racial variability in psychological consequences of illicit drug use. In addition, African Americans display a higher likelihood of being cocaine users compared to European Americans [86]. Analogously, the social and psychological context of marital dissolution have also been perceived to be contrasting in African and European Americans [87]. These inter-racial differences might account for non-significant interactions in African American population observed in this study.

Differences in genetics of AA and EA populations (e.g. large difference in minor allele frequencies of rs16969968 among these two ethnicities rendering this variant less informative in AAs) may account for the lack of observed interaction between *CHRNA5* and conscientiousness in AA participants. Such dissimilarity in gene x environment interaction has already been documented [75], [88].

In conclusion, findings of this study suggest that NEO-PI-R personality measures may play an important role in substance use disorders on both environmental (marriage) and genetic (*CHRNA5*) levels. Although the presented results should be interpreted as exploratory and in need of replication, they do add to a growing body of evidence that personality traits may not necessarily be the cause of drug addiction, but, in combination with other etiological factors, may influence its form or severity, as well as the development of co-occurringpsychopathologies.
